# Attitudes of older adult care workers towards older adults and their influencing factors in Western China

**DOI:** 10.3389/fpubh.2026.1829497

**Published:** 2026-06-29

**Authors:** Ling-ying Wang, Yu-jing Fang, Li Liu, Li Wang, Yan Dai

**Affiliations:** 1 Critical Care Medicine Department, West China Hospital, Sichuan University/West China School of Nursing, Sichuan University, Chengdu, China; 2 Chengdu Huaxi Health Care Vocational Skills Training School Co., Ltd., Chengdu, China; 3 Innovation Center of Nursing Research and Nursing Key Laboratory of Sichuan Province, West China Hospital, Sichuan University/West China School of Nursing, Sichuan University, Chengdu, China; 4 Nursing Department, West China Hospital, Sichuan University, Chengdu, China

**Keywords:** attitude toward older adults, care, older adult care worker, factor, nursing home

## Abstract

**Background:**

Older adult care workers, as frontline providers of daily care and spiritual comfort, have attitudes that directly affect care quality. However, existing research on aging attitudes has mainly focused on nursing students and clinical nurses, with a lack of specialized studies on older adult care workers.

**Objective:**

This study aimed to explore the current attitudes toward older adults among older adult care workers in Sichuan Province and identify their influencing factors using a quantitative approach, providing an objective basis for formulating targeted intervention programs.

**Method:**

A questionnaire survey was conducted among 400 older adult care workers from 42 nursing homes in Sichuan Province from June to August 2025. Data were analyzed using SPSS 26.0. An independent sample t-test or one-way ANOVA was used for univariate analysis, and multiple linear regression was applied to identify related factors.

**Results:**

A total of 301 valid questionnaires were recovered. The total score of KAOPS was 118.49 ± 17.022, with 92.7% of the participants showing a positive attitude toward older adults. Multiple linear regression analysis revealed that average monthly income, social support, marital status, experience of caring for older adults with agitated behaviors, and job burnout were associated with attitudes (all *p* ≤ 0.05).

**Conclusion:**

Older adult care workers in Sichuan Province generally hold positive attitudes toward older adults. Average monthly income, social support, marital status, experience with agitated behaviors among older adults, and job burnout have a significant impact on their attitudes. Targeted interventions (e.g., enhancing social support, reducing burnout) are needed to optimize their care attitudes and improve institutional older adults’ care quality.

## 1 Introduction

As of the end of 2024, China’s population aged 60 and above reached 310 million, accounting for 22.0% of the total population. The aging trend continues to accelerate (1). China is facing the triple challenges of rapid aging, the rise of empty-nest families, and the nuclearization of family structures. Against this backdrop, the traditional family-based older adults care model can no longer meet social needs. Consequently, institutional older adults care has become a key pillar of the older adults care service system (2). However, several challenges remain. These include improving service quality, meeting the diverse needs of older adults, and building a humanized care system (3). Statistical data show that from 2016 to 2020, the total number of older adults care service institutions and facilities nationwide surged from 116,000 to 329,000. Over the same period, the number of older adults care beds increased from 6.727 million to 8.21 million (4). Along with the rapid expansion of care infrastructure, the population receiving institutional care has also shown significant growth (5).

Older adult care workers, as professional service personnel who directly provide daily care and spiritual comfort to older adults (6), their service attitude directly affects the quality of care behavior. A positive service attitude is positively correlated with the endogenous motivation of caregivers, promote active care behaviors, and thus improve service quality; while a negative attitude may be related to a decline in the quality of life and subjective well-being of older adults, thereby hindering the process of healthy aging (6). Existing studies have shown that the level of caregivers’ attitudes towards older adults is an important variable in predicting the quality of care (7, 8). Therefore, systematically examining the current situation of their attitudes and influencing factors, and improving care awareness and service attitudes through targeted training, is of key significance for optimizing the quality of institutional older adults’ care services.

Literature analysis shows that current research on aging attitudes mostly focuses on nursing students and clinical nurses, and there is a lack of specialized research on older adult care workers. Although in recent years, many studies have been committed to building standardized training systems to improve the professional skills of caregivers, they generally ignore the predictive role of attitude factors on care behaviors. Ageism refers to stereotypes, prejudice, and discrimination against individuals based on age (9). The WHO Global Report on Ageism identifies negative attitudes toward older adults as a core form of ageism and stigma, which can impair care quality and the well-being of older adults (10). Based on this, carrying out systematic research on the aging attitudes of older adult care workers has important theoretical value and practical significance. This study adopts a quantitative research method, taking older adult care workers in Sichuan Province as the research object, aiming to explore the current situation of attitudes towards older adults among older adult care workers in Sichuan Province and its influencing factors, so as to provide an objective basis for formulating targeted intervention programs.

## 2 Methods

### 2.1 Study design and participants

#### 2.1.1 Study design

A questionnaire survey was conducted among older adult care workers in Sichuan Province, a typical representative region in Western China with a large aging population and diverse nursing home facilities, from June to August 2025. The target population was older adult care workers who provide direct daily care for older adults in nursing homes in Western China. The inclusion criteria were as follows: ① provided informed consent and voluntarily participated in the survey; ② aged ≥18 years; and ③ worked in a nursing home. The exclusion criteria were as follows: ① participants who declined to take part in the survey; ② working time of less than 6 months (to ensure sufficient exposure to care for older adults).

#### 2.1.2 Participants

A convenience sampling was used to recruit older adult care workers from forty-two nursing homes in Sichuan Province, China, as our study participants (*n* = 400). Employing SurveyStar, we distributed an online questionnaire. Ensuring adherence to principles of informed and voluntary consent, we provided comprehensive details about the study’s objectives and secured their agreement to participate. Then, two researchers double-checked the quality of the questionnaire and deleted those that violated the filling requirements or were completed within 180 s. In the pilot study, 30 individuals pre-filled the questionnaire, which took an average of over 180 s to complete. Consequently, questionnaires completed in under 180 s were deemed to have been answered carelessly. The 99 invalid questionnaires were due to refused individuals (*n* = 93), and 6 individuals completed within 180 s. A total of 301 participants were finally included in the study.

#### 2.1.3 Sample size

A *post hoc* power analysis was conducted using G*Power 3.1. The effect size *f*^2^ was 0.325 (based on *R*^2^ = 0.247), *α* = 0.05, and sample size = 301. The achieved statistical power was 0.999, exceeding the recommended threshold of 0.80, indicating that the sample size was sufficient.

### 2.2 Ethical approval

Approval for this study was granted by the Science and Ethics Committee of Sichuan University West China Hospital (Approval Number: 2025-1,124, the approval date was June 13, 2025, which was prior to data collection). The survey adhered to the principles outlined in the Declaration of Helsinki during its implementation.

### 2.3 Research methods

#### 2.3.1 General data

We utilized a self-developed questionnaire, which was developed based on literature review and expert consultation (*n* = 5 experts), to gather data on several factors, including gender, age, marital status, educational background, professional technical rank, religious affiliation, average monthly income, work conditions including working years, shift duration, resident-to-caregiver ratio, and night shift frequency, training background, work satisfaction (measured using a Likert scale with five response options ranging from “very dissatisfied” to “very satisfied”), on-the-job-training, and agitated behaviors [including verbal (attention-seeking, repetition, complaining, screaming, groaning, nonsense, unwarranted laughter, singing) and physical (hitting, kicking, pushing, scratching, tearing, cursing, grabbing, pacing, disrobing, exiting, handling things inappropriately, restlessness, repetitive mannerisms)] in older adults residents. A pilot study (*n* = 30) was conducted, and the content validity index (CVI) was 0.89. The general data questionnaire in this study was primarily used to collect objective background information. Content validity was ensured through expert assessment, while internal consistency testing was not employed.

#### 2.3.2 Social support

The score of social support was measured by the social support rating scale (SSRS) (11). Consists of three sub-domains, such as objective support, subjective support, and support utilization, its total scores range from 12 to 66, and higher scores indicate better social support. Cronbach’s alpha, split-half correlation coefficient, and test–retest correlation coefficient of SSRS were 0.821, 0.875, and 0.829, respectively, indicating high reliability (12). As a questionnaire to evaluate social support, SSRS has been widely accepted and used in epidemiological studies across China (12, 13).

#### 2.3.3 Job burnout

Job burnout were measured by the Chinese version of Maslach Burnout Inventory-General Survey (MBI-GS), translated by Li et al (14). The scale consists of three dimensions: (1) the emotional exhaustion dimension (consisting of five items); (2) the depersonalization dimension (consisting of four items); and (3) the low personal accomplishment dimension (consisting of six items). Each item is assessed using a seven-point Likert scale, ranging from 0 (never) to 6 (every day). The personal accomplishment dimension adopts a reverse scoring rule, while all other dimensions use positive scoring. The comprehensive score is calculated based on the weights of each dimension using the formula: Total Score = (Emotional Exhaustion×40%) + (Cynicism×30%) + (Reduced Professional Efficacy×30%). A higher score indicates a more severe level of burnout. To further determine the burnout level: (1) A comprehensive score of less than 1.5 is classified as negative (no burnout). (2) A score of 1.5 or higher but less than 3.5 is classified as mild to moderate burnout. (3) A score of 3.5 or higher is classified as severe burnout. In this study, all cases with a comprehensive score of 1.5 or higher were uniformly identified as having burnout for analysis. The Cronbach’s *α* coefficient of the scale is 0.88, and the Cronbach’s *α* coefficients of each sub-dimension are 0.96, 0.95, and 0.95, respectively (15).

#### 2.3.4 Attitudes toward older adults

Attitudes toward older adults were measured by the Chinese version of the Attitudes Toward Old People Scale. Kogan developed the Kogan’s Attitudes Toward Old People Scale (KAOPS) in 1961 to measure the attitude tendencies of clinical medical staff and medical students towards older adults (16). In 2012, this scale was sinicized by Zhao (17). The total scale includes 32 items. The Cronbach’s *α* coefficients of the total scale, positive subscale, and negative subscale are 0.85, 0.84, and 0.81, respectively. With 96 points as the cutoff, a higher score indicates a more positive attitude. In the present study, the Cronbach’s α of the scale was 0.81, indicating excellent internal consistency.

### 2.4 Data analysis

Data organization was accomplished using Microsoft Excel, while analysis was conducted using SPSS version 26. First, we conducted Harman’s single-factor test to examine common method bias in the collected data. Enumeration data are expressed as the number of cases. Measurement data that pass the normality test and homogeneity of variance test are expressed as mean ± standard deviation (x̄±s). Normality was examined using the Shapiro–Wilk test. The KAOPS total score was normally distributed (W = 0.949, *p* < 0.001), with skewness = 0.960 and kurtosis = 2.004. The MBI-GS total score was also normally distributed (W = 0.938, *p* < 0.001, skewness = 0.762, kurtosis = 0.401). The Shapiro–Wilk test indicated that the KAOPS and MBI-GS total scores did not follow a normal distribution (*p* < 0.05). However, the absolute values of skewness and kurtosis were within acceptable ranges, and the Q-Q plot showed that the data points were generally distributed along the diagonal line. Based on the central limit theorem (sample size *N* = 301), subsequent analyses were conducted using parametric tests. Other categorical variables did not meet normality assumptions, and non-parametric tests were used accordingly.

Independent sample *t*-test or one-way analysis of variance (ANOVA) is used for comparison between groups, with Bonferroni correction for multiple comparisons. Multiple linear regression analysis was performed using the Enter method, in which variables with statistical significance in the univariate analysis were simultaneously entered into the model to control for potential confounding factors. Therefore, individual variables that did not reach statistical significance were still retained in the final model. Regression assumptions were fully checked: multicollinearity was assessed using variance inflation factors (VIF), with all VIF < 2.0 indicating no multicollinearity; the Durbin–Watson value was 1.937, indicating no autocorrelation; and residuals were normally distributed. A *p* value ≤ 0.05 is considered statistically significant. Effect sizes were evaluated using Cohen’s d for *t*-tests and η^2^ for ANOVA. According to the criteria proposed by Cohen (18), Cohen’s d values of 0.2, 0.5, and 0.8 represent small, medium, and large effects, respectively; η^2^ values of 0.01, 0.06, and 0.14 represent small, medium, and large effects, respectively. For regression analysis, adjusted R^2^ is reported as the effect size for the model.

## 3 Results

### 3.1 Common method bias

Common method bias was evaluated using Harman’s single-factor test. The results indicated that nine factors had characteristic roots of >1. The first unrotated factor accounted for 16.17% of the total variance, which was less than the 40% threshold (19), suggesting no substantial common method bias.

### 3.2 Descriptive statistics and correlation analysis

A total of 400 older adult care workers from Sichuan Province were invited to participate in this.

study, and 301 valid questionnaires were recovered. The basic characteristics of the sample are shown in Table 1. There were 265 females, accounting for 88.0%; the age group of 30–39 years old accounted for the highest proportion (36.9%); the majority were married (81.7%); in terms of education level, those with a bachelor’s degree or above accounted for 29.6%; regarding professional titles, 39.5% of the care workers had no professional title; only 6.0% had religious beliefs; and the level of social support was mainly low to moderate (51.5%).

**Table 1:** Univariate analysis of general data and scores of the aging attitude scale among older adult care workers (x̄±s, scores).

Variable	Number (%)	Total score of older adults attitude scale	*t/F*	*p* (Effect size)
Gender				
Male	36 (12.0)	118.97 ± 21.30	0.181	0.856 (*d* = 0.03)
Female	265 (88.0)	118.42 ± 16.40		
Age			2.005	0.113 (η^2^ = 0.02)
<30	53 (17.6)	121.70 ± 16.85		
30–39	111 (36.9)	119.03 ± 16.23		
40–49	101 (33.6)	118.22 ± 18.02		
>50	36 (12.0)	112.86 ± 16.03		
Marital status			−2.385	0.018 (*d* = −0.36)
Married	246 (81.7)	117.39 ± 15.36		
Non-married (single/divorce)	55 (18.3)	123.40 ± 17.58		
Education			1.636	0.181(η^2^ = 0.02)
Primary school/Junior high school	69 (22.9)	118.54 ± 15.36		
Senior high school/vocational school	59 (19.6)	118.20 ± 18.49		
Junior college	84 (27.9)	115.63 ± 14.39		
Undergraduate/Postgraduate	89 (29.6)	121.34 ± 19.21		
Professional technical rank			4.034	0.019 (η^2^ = 0.03)
None	119 (39.5)	118.14 ± 18.22		
Primary	69 (22.9)	123.23 ± 16.47		
Intermediate	113 (37.5)	115.96 ± 15.54		
Religious			−1.232	0.219 (*d* = 0.30)
Have	18 (6.0)	123.28 ± 13.83		
None	283 (94.0)	118.18 ± 17.18		
Social support			−3.090	0.002 (*d* = −0.36)
Low/Median	155 (51.5)	115.59 ± 14.08		
High	146 (48.5)	121.57 ± 19.25		
Work satisfaction			25.936	<0.001 (η^2^ = 0.15)
Neutral/dissatisfied	60 (19.9)	116.17 ± 13.07		
Satisfied	167 (55.5)	114.28 ± 14.54		
Very satisfied	74 (24.6)	129.88 ± 19.88		
On-the-job training			2.306	0.031 (*d* = −0.43)
Have	282 (93.7)	118.95 ± 17.18		
None	19 (6.3)	111.68 ± 12.98		
Working years			0.807	0.491 (η^2^ = 0.01)
≤5	157 (52.2)	119.10 ± 15.92		
6–10	87 (28.9)	118.39 ± 18.75		
11–20	45 (15.0)	118.49 ± 18.46		
>20	12 (4.0)	111.17 ± 11.49		
Daily working hours			0.194	0.823 (η^2^ < 0.01)
<8	76 (25.2)	119.53 ± 16.31		
8–10	155 (51.5)	118.05 ± 17.10		
>10	70 (23.3)	118.33 ± 17.79		
Resident-to-caregiver ratio			2.541	0.057 (η^2^ = 0.03)
1–5	145 (48.2)	121.24 ± 18.34		
6–10	41 (13.6)	114.98 ± 18.21		
11–15	16 (5.3)	116.75 ± 15.70		
>15	99 (32.9)	116.19 ± 14.06		
Monthly night shift frequency			0.298	0.827 (η^2^ < 0.01)
0	71 (23.6)	119.58 ± 19.07		
1–4	71 (23.6)	118.49 ± 15.63		
5–8	74 (24.6)	117.00 ± 15.30		
>8	85 (28.2)	118.87 ± 17.94		
Average monthly income (¥)			5.294	0.001 (η^2^ = 0.03)
<4,000	146 (48.5)	115.63 ± 15.55		
4,000–6,000	103 (34.2)	118.60 ± 14.31		
6,000–8,000	31 (10.3)	127.13 ± 25.16		
≥8,000	21 (7.0)	125.05 ± 19.49		
Agitated behaviors in older adults residents			−3.052	0.002 (*d* = −0.35)
Have	156 (51.8)	115.64 ± 15.30		
None	145 (48.2)	121.55 ± 18.26		
Job burnout				
Have	249 (82.7)	115.27 ± 13.82	−5.863	**<0.001** (*d* = −1.20)
None	52 (17.3)	133.88 ± 22.00		

For effect sizes, Cohen’s d is reported for t-tests, and η^2^ (eta-squared) is reported for ANOVA. Bonferroni correction was applied for multiple comparisons.

In terms of work characteristics, over half (55.5%) of older adult care workers were satisfied with their work, and another 24.6% were very satisfied with their work, with the combined proportion reaching 80.1%; the vast majority (93.7%) had received on-the-job training; the majority had 1–5 years of work experience (52.2%); those with an average daily working hour of 8–10 h accounted for the highest proportion (51.5%); the number of older adults cared for was mostly 1–5 (48.2%); 28.2% of the care workers worked more than 8 night shifts per month; 48.5% had an average monthly income of less than 4,000 yuan; 51.8% of the care workers took care of older adults with agitated behaviors. In addition, the total mean score of job burnout is 6.12 ± 4.726, and 82.7% of the care workers showed signs of job burnout.

The scores of older adult care workers in Sichuan Province on their attitudes towards older adults range from 73 to 189. The total mean score of KAOP is 118.49 ± 17.022, which is higher than the median score of 96. With 96 as the critical value to distinguish the level of older adult care workers’ attitudes towards older adults, the results show that 20 older adult care workers have a tendency towards negative attitudes, accounting for 6.6%; 2 have neutral attitudes, accounting for 0.7%; and 279 have a tendency towards positive attitudes, accounting for 92.7%. There are statistically significant differences in the scores of the Aging Attitude Scale among older adult care workers with different marital status, social support, job satisfaction, on-the-job training, experience of taking care of older adults with agitated behaviors, and job burnout (*p* ≤ 0.05), as shown in Table 1.

Multiple linear regression analysis was performed with the total score of the Aging Attitude Scale as the dependent variable and the variables with statistical significance in the univariate analysis as independent variables (marital status, professional technical rank, social support, work satisfaction, on-the-job training, average monthly income, agitated behaviors in older adult residents, and job burnout. The job burnout was analyzed using continuous total scores instead of dichotomization to retain full information). The results showed that average monthly income, social support, marital status, experience of caring for older adults with agitated behaviors, and job burnout scores were related to care workers’ attitudes towards older adults (all *p* ≤ 0.05). The 95% confidence intervals (95% CI) for these significant variables did not cross zero, confirming the stability and reliability of the estimated effects. Non-significant variables (on-the-job training, night shift frequency, and work satisfaction) showed 95% CI crossing zero, consistent with their non-significant *p*-values. Details are shown in Table 2.

**Table 2:** Multiple linear regression analysis of influencing factors on older adult care workers’ attitudes towards older adults.

Variables	Unstandardized coefficients (B)	95% CI	*p*
Constant	90.211	(74.544, 105.877)	<0.001
Professional technical rank	−0.917	(−2.939, 1.104)	0.373
On-the-job training	3.427	(−3.644, 10.498)	0.341
Average monthly income	3.683	(1.761, 5.606)	**<0.001**
Work satisfaction	2.243	(−0.600, 5.086)	0.122
Social support	5.077	(1.253, 8.901)	**0.009**
Marital status	9.979	(5.246, 14.712)	**<0.001**
Agitated behaviors in older adult residents	−4.852	(−8.386, −1.319)	**0.007**
Job burnout	−1.047	(−1.445, −0.649)	**<0.001**

*R*^2^ = 0.267, ∆ *R*^2^ = 0.247; *F* = 13.277, *p* < 0.001.

## 4 Discussion

Population aging is the main feature of the current changes in China’s population structure. Improving the care security and service system for older adults is an important part of actively responding to population aging (20). As the main service providers in older adults care institutions, understanding the current situation of older adult care workers’ attitudes towards older adults is of great significance for improving the care service system and enhancing the quality of care for older adults.

The results of this study showed that the attitude scores of 301 older adult care workers towards older adults ranged from 71 to 189, with the total score of attitudes towards older adults being (118.49 ± 17.022). With 96 as the critical value to distinguish the attitude levels of older adult care workers towards older adults, the results showed that 6.6% of older adult care workers had a tendency to negative attitudes, 0.7% had neutral attitudes, and 92.7% had a tendency to positive attitudes. It can be seen that the attitude level of older adult care workers towards older adults in this study was slightly positive, which was similar to the results of most domestic studies (21–23). This may be related to China’s traditional ideas of “filial piety” and “respecting and caring for older adults”, as well as the similar characteristics of older adult care workers in China, such as their age group, educational experience, and most of them coming from rural areas, and the fact that older adults care education has gradually received attention in recent years. Although most care workers in this study reported positive attitudes toward older adults, 6.6% still showed negative attitudes, which may reflect ageism, stigma, and negative stereotypes toward older adults. Ageism, as defined by Butler (9) and emphasized in the WHO Global Report on Ageism (10), involves prejudice and discrimination based on age and can undermine person-centered care. Therefore, attention should be paid to this subgroup in future training and management to reduce ageist beliefs and stigma. The high proportion of positive attitudes (92.7%) should be interpreted cautiously, as this result may be affected by social desirability bias, a common limitation in self-reported studies. Participants may tend to provide socially desirable responses rather than their genuine views, which may have led to an overestimation of positive attitudes. In addition, while positive attitudes toward older adults are generally considered important for care services, this study only measured care workers’ attitudes and did not assess actual care behaviors, objective care quality, or care outcomes. Attitude does not equate to actual behavior, and no causal inference can be made that positive attitudes directly lead to better care. This should be recognized when interpreting the findings.

The generally positive attitudes observed in this study can also be deeply explained by traditional Chinese cultural values, particularly Confucian filial piety (24). Rooted in long-standing social norms, filial piety emphasizes respect, care, and emotional support for older adults, which are widely internalized as moral and social obligations. These cultural values shape care workers’ empathy, sense of responsibility, and person-centered care orientation, contributing to their positive attitudes toward older adults. Unlike Western contexts that may emphasize individual autonomy, Chinese care workers are more likely to view caring for older adults as a socially valued and morally meaningful practice, which further reinforces positive attitudes.

A study by López-Hernández et al. (25) found that nursing students in Spain generally held positive attitudes toward older adults. Mohammadi et al. (26) reported that professional nurses providing care for older adults exhibited more positive attitudes than nursing students, a finding largely consistent with that of Pekcetn (27). Loi et al. (28) indicated that caregivers working in Australian aged care facilities held overall positive attitudes toward older adults. Similarly, Doherty et al. (29) observed positive attitudes toward older adults among nurses, care workers, and students in Ireland. In contrast, Luchesi et al. (30) concluded that caregivers of older adults in Brazil held neutral attitudes, while Cheon et al. (31) also reported neutral attitudes toward older adults among Korean nurses and nurse aides. Compared with the above international findings, the present study revealed higher attitude scores among caregivers of older adults, suggesting that caregivers in Sichuan Province hold positive attitudes toward older adults. Several factors may account for this result. First, the majority of participants in this study were aged 30–39 years, married, and holding a bachelor’s degree or above, a group likely characterized by stronger professional responsibility and identity. Second, relatively high levels of job satisfaction may have contributed to more positive perceptions of older adults. Third, participation in on-the-job training aimed at improving care competencies may have strengthened caregivers’ confidence in practice and further fostered positive attitudes.

This study found that monthly income is an important factor influencing older adult care workers’ attitudes toward older adults, with those having higher monthly income demonstrating more positive attitudes. This finding is consistent with multiple domestic and international studies. Yuan et al.’s survey of older adult care workers in Changsha showed that salary income was a significant factor affecting caregivers’ attitudes toward older adults (*p* < 0.05) (32). Cui et al. (33) empirical study based on older adult care workers in Shanghai also found that caregivers with a monthly income of 5,000–6,999 yuan had higher satisfaction with their salary and treatment compared to those with income below 3,000 yuan, and salary satisfaction was positively correlated with work enthusiasm and care attitudes. International research supports this finding as well. The OECD report “Who Cares? Attracting and Retaining Elderly Care Workers” indicates that long-term care workers earn significantly less than those working in hospitals in similar occupations (median hourly wage of €9 vs. €14 in European countries), and low pay is a major reason for high turnover rates and low professional identity in the care sector (34). Evidence from the United States and France shows that wage increases in long-term care are associated with greater recruitment of workers, longer tenure, and lower turnover. Mehdi et al.’s comparative study of healthcare workers in Germany and Canada also demonstrated that income level is closely associated with employment stability and career development opportunities for care workers, which in turn is significantly related to their positive attitudes toward care recipients (35). Several factors may explain this relationship. First, higher economic income can alleviate caregivers’ financial stress, enabling them to devote themselves to care work with greater peace of mind and a more positive attitude. Second, monthly income reflects, to some extent, the level of societal recognition for the care profession; the sense of professional respect associated with higher income helps strengthen caregivers’ professional identity and self-efficacy. Third, caregivers with higher income often hold more responsible positions or have richer experience in their institutions, which may contribute to a deeper understanding of older adult care and more positive attitudes. Therefore, managers of older adult care institutions and policymakers should pay attention to the impact of compensation on caregivers’ attitudes, appropriately raise salary levels, and establish reasonable salary incentive mechanisms to enhance caregivers’ job satisfaction and positive attitudes toward older adults, thereby improving the quality of older adult care services.

This study found that social support shows a positive correlation with the attitudes of older adult caregivers, which is consistent with the conclusions of mainstream research at home and abroad. For example, a survey by Sun et al. (36) found that core competence is positively correlated with social support and its various dimensions. Higher social support is positively correlated with the core competence of older adult caregivers, thereby promoting a more positive attitude towards caregiving. A survey on caregivers in older adults care institutions in Nantong also showed that caregivers with higher social support levels performed better in terms of job satisfaction and emotional stability (37), which was highly consistent with the results of this study, confirming the cross-regional consistent role of social support. A Swedish study on family caregivers of dementia patients found that providing social support through mobile health (mHealth) technology (such as online psychological counseling and care skills training) could significantly reduce caregivers’ depressive symptoms and enhance their patience and empathy for dementia patients (38); a study in Egypt showed that the social support network (such as family and community) of older people with chronic diseases could buffer their sense of psychological burden and indirectly improve caregivers’ care experience (39), which further supports the universality of the conclusions of this study.

Older adults’ care work is characterized by a high workload and high emotional investment. Caregivers often experience negative emotions such as anxiety and burnout due to the complex care needs of older adults and frequent emergencies. Social support provides an “external buffer” to reduce the accumulation and transmission of negative emotions. Studies have reported that emotional support from family members can help caregivers quickly relieve negative emotions and prevent the conversion of fatigue into indifference towards older adults (40); instrumental support among colleagues can reduce work intensity, enabling caregivers to have more energy to focus on the emotional needs of older adults (41). It is worth noting that social support in this study is mainly at a low/moderate level (51.5%), reflecting that there is still room for improvement in the current social support situation for older adult caregivers in China. Under the current development of institutional older adult care in China, older adult care workers often face heavy workloads, irregular shifts, and limited opportunities for social interaction. Meanwhile, social recognition and policy support for this occupational group are still insufficient, and family and community support systems are not fully established. The relatively low level of social support may reduce care workers’ psychological resilience and professional identity, which in turn is associated with their attitudes toward the older adults. This finding highlights the urgency to build a multi-dimensional social support system suitable for Chinese older adult care workers, including institutional support, family support, and social recognition.

This study found that non-married older adults caregivers have a more positive attitude towards older adults, which is consistent with the findings of Nordic studies (42), unmarried or divorced caregivers have a higher work commitment due to lighter family burdens. A possible explanation is that non-married caregivers (such as the unmarried or divorced) may have more time and energy to devote to their work and are less constrained by family responsibilities, thus showing a higher degree of concentration and patience when caring for older adults. In addition, married caregivers may be more prone to occupational burnout due to family pressures (such as child rearing, spousal relationships, etc.), which in turn related to their care attitudes. However, a survey conducted by Yuan et al. (32) in Changsha pointed out that married older adult caregivers scored significantly higher in their attitudes towards older adults than unmarried ones. Another study further indicated that married caregivers have a higher level of knowledge about aging, which helps them form a more professional care attitude (43). This inconsistency may be attributed to several study-specific factors. First, non-married caregivers in our sample were predominantly young adults with relatively high educational levels, which may have endowed them with more comprehensive knowledge of aging processes and age-friendly concepts, thereby fostering more positive attitudes. Second, non-married caregivers often experience less work–family conflict and fewer family-related burdens, allowing them to dedicate more emotional energy and professional focus to their caregiving roles. Furthermore, the supportive organizational environment and strong peer relationships within the care team may have further enhanced their positive perceptions of older adults. This finding underscores that marital status is not the sole determinant of attitudes toward older adults; instead, it interacts with other individual and contextual factors to shape care workers’ attitudes. Future research can further explore the interaction between marital status and work–family conflict to more comprehensively understand this phenomenon.

Studies have shown that older adult caregivers tend to hold more negative attitudes when the care recipients exhibit agitated behaviors, and this finding is highly consistent with a study by The Hong Kong Polytechnic University on aggressive behaviors among nursing home residents. The study revealed that caregivers’ exposure to aggressive behaviors from older adults (such as verbal or physical attacks) significantly increases their level of occupational burnout and reduces their willingness to provide care (44). Agitated behaviors (such as resisting care or using aggressive language) not only increase caregivers’ work pressure but also may trigger negative emotions like anger or frustration, thereby undermining their positive attitudes. Almost half of all nursing home residents living with moderate or severe Alzheimer’s disease and related dementias experience clinically meaningful agitation (45). A survey by McCreedy et al. (46) found that agitated behaviors of older people with dementia were significantly correlated with caregivers’ emotional burden and social burden. When caregivers face such behaviors for a long time, they are prone to feelings of frustration, which in turn affects their patience and empathy towards older adults. Therefore, it is imperative to develop and implement targeted training programs, such as specialized courses on dementia care, evidence-based de-escalation techniques, non-violent communication skills, and stress management strategies. Regular workshops and hands-on simulation exercises can also enhance care workers’ practical competencies, reduce perceived stress, and foster more positive attitudes toward older adults experiencing agitated behaviors.

This study found that older adult caregivers experiencing occupational burnout hold more negative attitudes towards older adults, which is consistent with the results of multiple domestic and international studies. For example, Zhou et al. (47) found that caregivers with severe occupational burnout tend to adopt a mechanized care model, reducing emotional interaction with older adults and affecting service quality. Occupational burnout is usually caused by factors such as long-term work pressure, low salary, and high workload, leading to emotional exhaustion, depersonalization (i.e., indifference or alienation towards older adults), and reduced personal sense of accomplishment. A study in Hong Kong also found that burned-out caregivers are more likely to develop negative stereotypes about older adults, such as perceiving them as “stubborn” or “difficult to communicate with,” and are more prone to adopting communication avoidance strategies (44). A review on long-term care institutions found that burned-out caregivers have lower tolerance for aggressive behaviors of older adults and are more likely to feel frustrated (48). Given the cross-sectional design, the directionality of this relationship cannot be determined. However, a bidirectional association is biologically and psychologically plausible. On the one hand, severe burnout may lead to emotional exhaustion and cynicism, which are associated with more negative attitudes toward care recipients. On the other hand, pre-existing negative attitudes toward older adults may reduce job satisfaction and increase emotional distress, thereby further elevating the risk of burnout. Longitudinal or cross-lagged panel studies are needed to clarify the temporal order and causal direction between these two variables. At the same time, institutional managers should pay attention to the mental health of caregivers and reduce the risk of occupational burnout by optimizing work schedules, increasing salaries, and providing psychological counseling.

In the regression analysis, all significant factors showed 95% CIs that did not include zero, which further verified the reliability and stability of these effects. The confidence intervals reflected the estimation precision of each factor’s relate on attitudes toward the older adults, supporting that average monthly income, social support, marital status, agitated behaviors, and job burnout were robust related factors. In addition, the multiple linear regression model showed an adjusted *R*^2^ value of 0.247, indicating that the included variables (average monthly income, social support, marital status, agitated behaviors, and job burnout) explained approximately 24.7% of the total variance in care workers’ attitudes toward the older adults. This modest explanatory power is relatively common in cross-sectional observational studies of care workers, suggesting that other unmeasured factors may also relate to attitudes toward older adults, such as personality traits, aging-related knowledge, institutional management culture, work–family conflict, and social attitudes toward aging. This study only employed a quantitative questionnaire survey and did not collect qualitative data. Future research could adopt a mixed-methods design, incorporating qualitative data to more comprehensively explain the underlying reasons for caregivers’ attitudes. Additionally, future studies may adopt a more comprehensive variable framework or use longitudinal designs to improve the explanatory power of regression models and explore causal pathways more deeply.

Last but not least, older adult care workers in Sichuan Province held positive attitudes toward older adults (92.7%) is comparable and consistent with international studies conducted among other occupational groups. A survey by Solak et al. (49) among 283 public sector employees in Turkey showed that participants held positive attitudes toward older adults because “respecting older adults is an important part of cultural heritage,” yet they simultaneously held certain discriminatory views toward older employees. This finding echoes the cultural explanation of the present study—traditional Chinese values of “filial piety” and “respecting and caring for the older adults” similarly shape older adult care workers’ positive attitudes. However, Solak et al. (49) study also reveals an important contradiction: general respect for older adults and specific discrimination against older employees can coexist. This suggests that although the present study found care workers to hold positive attitudes toward older adults, one should not assume that these positive attitudes naturally extend to evaluations of older colleagues or older employees. A comparative study by Gallagher et al. (50) of personnel in acute and long-term care institutions found that assistants and maintenance staff held significantly more negative attitudes than nurses, and that negative attitudes were associated with lower educational levels. This finding is highly consistent with the regression analysis results of the present study (education being a significant positive influencing factor on attitude scores, *β* = 0.186, *p* = 0.002), further supporting the key role of educational level in shaping positive attitudes. A qualitative systematic review by Jeyasingam et al. (51) pointed out that healthcare professionals’ attitudes toward aging are influenced by professional experience and systemic factors, with physicians tending to emphasize complexity and dependency, while nurses focus more on care burden. The review also identified an important theme—the “systemic context” of caregivers’ attitudes toward older adults, including factors such as resource shortages, burnout, and compassion fatigue. This provides theoretical support for the “unmeasured influencing factors (e.g., institutional management level, organizational support)” raised in the discussion of the present study. Furthermore, an audit experiment study on older adults care in the Netherlands and Belgium found that activating prosocial stereotypes (public sector employees being perceived as “caring and helpful”) could improve the friendliness of the service process but did not necessarily enhance response rates or the objective quality of information provided (52). This study suggests that the relationship between positive attitudes and care behaviors is not a direct causal chain, which is consistent with the emphasis in the limitations section of the present study that “attitudes do not equate to actual care behaviors.

## 5 Strengths and limitations

This study fills the research gap by focusing specifically on older adult care workers, a group understudied in previous aging attitude research. It quantitatively analyzes multiple related factors (e.g., job burnout, social support) in a systematic manner, providing empirical evidence for understanding the attitudinal mechanisms of this professional group. Although the questionnaire was administered anonymously to reduce social desirability effects, this bias cannot be completely eliminated. Future research may combine implicit attitude measures (e.g., implicit association tests) or observational methods to more objectively assess caregivers’ true attitudes.

The sample was restricted to Sichuan Province, limiting the generalizability of results to other regions. Although Sichuan Province is a typical province in western China, with a large workforce of older adult care workers and diverse types of care institutions, there are significant differences across provinces in terms of economic development level, policy support, and the construction of older adult care service systems. Therefore, the generalization of the findings of this study to the entire western China region should be made with caution. Future research should conduct multicenter, cross-provincial, large-sample surveys to validate the generalizability of the conclusions. The cross-sectional design cannot establish causal relationships between influencing factors and attitudes. Because an anonymous online questionnaire survey was employed, which may have excluded older adult care workers who are less familiar with electronic devices or are older, demographic information on those who refused to participate or did not complete the questionnaire could not be obtained, which may introduce certain selection bias. Future studies should adopt a mixed online and offline survey approach to more comprehensively cover caregivers with diverse characteristics.

## 6 Conclusion

Older adult care workers in Sichuan Province have predominantly positive attitudes toward older adults. Average monthly income, social support, marital status, experience with agitated older individuals, and job burnout are critical factors associated with their attitudes, highlighting the need for strategies to strengthen social support networks, mitigate burnout, and provide training for managing agitated behaviors in older adults. These measures tends to be higher care workers’ positive attitudes, thereby improving the quality of institutional older adults care services.

## Data Availability

The raw data supporting the conclusions of this article will be made available by the authors, without undue reservation.
